# A Case Study of Nitrogen Saturation in Western U.S. Forests

**DOI:** 10.1100/tsw.2001.280

**Published:** 2001-11-08

**Authors:** Mark E. Fenn, Mark A. Poth

**Affiliations:** USDA Forest Service, Pacific Southwest Research Station, Forest Fire Laboratory, Riverside, California 92507, USA

**Keywords:** nitrogen saturation, California, western forests, mixed conifer forests, nitrate, nitrification, nitrogen mineralization, trace gas emissions, nitric oxide, nitrogen excess, nitrogen deposition, soil solution

## Abstract

Virtually complete nitrification of the available ammonium in soil and nitrification activity in the forest floor are important factors predisposing forests in the San Bernardino Mountains of southern California to nitrogen (N) saturation. As a result, inorganic N in the soil solution is dominated by nitrate. High nitrification rates also generate elevated nitric oxide (NO) emissions from soil. High-base cation saturation of these soils means that soil calcium depletion or effects associated with soil acidification are not an immediate risk for forest health as has been postulated for mesic forests in the eastern U.S. Physiological disturbance (e.g., altered carbon [C] cycling, reduced fine root biomass, premature needle abscission) of ozone-sensitive ponderosa pine trees exposed to high N deposition and high ozone levels appear to be the greater threat to forest sustainability. However, N deposition appears to offset the aboveground growth depression effects of ozone exposure. High nitrification activity reported for many western ecosystems suggests that with chronic N inputs these systems are prone to N saturation and hydrologic and gaseous losses of N. High runoff during the winter wet season in California forests under a Mediterranean climate may further predispose these watersheds to high nitrate leachate losses. After 4 years of N fertilization at a severely N saturated site in the San Bernardino Mountains, bole growth unexpectedly increased. Reduced C allocation below- ground at this site, presumably in response to ozone or N or both pollutants, may enhance the bole growth response to added N.

### INTRODUCTION

Aber et al.[1] proposed a conceptual model of forest responses to chronic nitrogen (N) inputs based on early studies from forests of the northeastern U.S. The syndrome of effects in this model is commonly referred to as N saturation. In this model it is hypothesized that over time chronic N inputs will result in progressively more severe symptoms of N saturation. For descriptive purposes, four stages were highlighted along the N saturation continuum, with stage 0 representing the normally N-limited condition. In stage 1 the incipient signs of N enrichment of the forest systems are observed as increased N concentrations in foliage and nitrification rates begin to increase. In stage 2 the forest can no longer retain all of the available soil N, and elevated hydrologic nitrate export is clearly evident. Gaseous emissions of N (e.g., $N_2O$, NO) from soil may also increase as a result of excess available inorganic N in soil. Stage 3 signifies decreased-net primary productivity or even forest decline. A similar description has been put forth of the four stages of N saturation based on temporal trends of nitrate export from forested watersheds[2].

Fenn et al.[3] proposed a modified conceptual model for California forests based on the climatic and edaphic conditions prevalent in this Mediterranean climate and based on what was known of the N cycling characteristics of these summer-dry ecosystems. The principal characteristics of this so-called "Western N Saturation Hypothesis" include active nitrification in soil and litter during all stages of N saturation, elevated emissions of nitric oxide (NO) from soil in sites with chronic N deposition, and temporal asynchrony between the major period of hydrologic fluxes (winter and early spring) and the period of greatest biotic N demand (late spring and early summer). As a result of these factors, it was hypothesized that forests with a Mediterranean climate and with chronic N deposition, such as in southern California, are highly prone to N losses from the ecosystem. In essence, the N cycle shifts from a relatively conservative or closed cycle in between fire events to an open or "leaky" N cycle.

The Western model also hypothesizes that California forests are prone to develop the symptoms of stage 2 of N saturation, but that stage 3 is less likely to occur in these systems compared to the mesic forests of Europe and the eastern U.S. Stage 3 in these northern temperate forests is presumably most likely to occur as a result of chemical changes such as calcium depletion, soil acidification, and mobilization of inorganic aluminum in soil[4]. Because mid- and low-elevation California forests have soils with high-base saturation, and soils which are less acidic and weathered than in northern Europe and in the northeastern U.S., there seems to be little risk of forest health effects related to soil acidification and cation depletion. However, stage 3 might occur if N excess were to predispose trees to other biotic or abiotic stresses. Pine trees affected by the combined effects of high ozone exposure and N deposition in the San Bernardino Mountains (SBM) do have greatly reduced fine root biomass, reduced C allocation belowground, and altered aboveground phenology[5,6]. However, it is not yet known how much of the observed physiological responses are due to ozone or to increased available N. The long-term consequences of the combined effects of ozone and N deposition for forest sustainability are also uncertain[7].

The western end of the SBM is clearly at stage 2 of the N saturation model as evidenced by high nitrate export in streamwater and elevated NO fluxes from soil[8,9]. Nitrate concentrations from streams in the western SBM and from chaparral watersheds in the San Gabriel Mountains, also in the Los Angeles Air Basin, are the highest reported for wildland watersheds in North America[8,10]. Previous studies in the SBM suggested that nitrification is a key process controlling N mobilization, because the primary end products of nitrification in these soils are nitrate and NO, both of which are readily lost from the forest in hydrologic outflows or as gaseous emissions from the forest floor. Therefore, the primary objectives of this study were to measure and compare rates of net N mineralization, net nitrification, and NO fluxes at an N-saturated and -limited site in the SBM. Long-term N fertilization treatments were also initiated at an N-limited and -saturated site in 1997 to further determine ecosystem responses to N inputs. It was hypothesized that tree growth would increase after fertilization in the eastern SBM low-deposition study site, but not in the study plots at the western high-deposition site. We also hypothesized that N fertilization at the low deposition site would induce N-saturation symptoms, evidenced as leached nitrate (data not presented in this paper) and elevated fluxes of NO.

### EXPERIMENTAL METHODS/PROCEDURES

#### Study Sites

The SBM are located ca. 70 km east of Los Angeles. This research was done at two main research sites: Camp Paivika (CP) is a high pollution site on the western end of the air pollution gradient in the SBM, and Barton Flats (BF) and Camp Osceola (CAO) are relatively low-deposition sites 42 to 46 km to the east of CP[11]. Elevation of the two sites is 1580 m at CP and 1945 m at Barton Flats, which is adjacent to CAO. Estimated annual precipitation at CP is 987 and 608 mm year$^{-1}$ at BF[8]. Average annual temperatures are 12.9°C at CP and 10.6°C at BF. Annual N deposition inputs are estimated to be 30 to 40 kg ha$^{-1}$ at CP and 4 to 8 kg ha$^{-1}$ at CAO[11,12].

Dominant overstory species at CP are ponderosa pine (*Pinus ponderosa* Laws.), California black oak (*Quercus kelloggi* Newb.), incense cedar (*Calocedrus decurrens* [Torr.], Florin.), and a minor component of sugar pine (*P. lambertiana* Dougl.). Bracken fern (*Pteridium aquilinum* var. *pubescens* Underw.) dominates the understory at CP. The overstory at BF/CAO is predominantly a mixture of ponderosa and the closely related Jeffrey pine (*Pinus jeffreyi* Grev. and Balf.), California black oak, and white fir (*Abies concolor* Gord. and Glend.).

#### Fertilizer Treatments and Tree Diameter Growth

At each of these two main study sites (CP and CAO), nine study plots were established in 1997 in a randomized block design. Each of three blocks consisted of an unfertilized control plot and a plot receiving 50 or 150 kg N ha$^{-1}$ year$^{-1}$ as a fertilization treatment. Fertilizer was applied annually on an individual tree basis (to an area circumscribed by the drip line and a 4-m diameter zone beyond the drip zone) beginning in 1997. Fertilizer was applied in winter or early spring as a slow release granular organic urea formulation. Each plot included at least 20 ponderosa or Jeffrey pine trees and 20 California black oak trees. Growth responses to N fertilization were evaluated by periodic measurements of bole diameter (diameter at breast height, or dbh). Data on bole diameter increase since the start of fertilization 4 years ago will be presented (from dbh measurements taken in the summer of 2001).

#### N Mineralization and Nitrification Assays and Soil Solution Concentrations

Annual net N mineralization and net nitrification rates were determined monthly for the forest floor (L, F, and H horizons combined) and the upper 10 cm of mineral soil using the soil tube method of *in situ* incubation[13]. Subsamples of the initial (time 0) and incubated litter and soil samples (after ca. 30 day) were extracted with 2*N* KCl and analyzed for $NH_4^+$ and $NO_3^-$ with a Technicon Autoanalyzer. From these analyses, net monthly ammonium and nitrate production were calculated. Net N mineralization (total of ammonium + nitrate minus the amount at time 0)

and net nitrification rates determined for each incubation period during the year were totaled to determine annual rates[14]. Soil solution concentrations of $NH_4^+$ and $NO_3^-$ were determined from samples collected on four dates in 1999 with the centrifugation method[9].

### Trace Gas Measurements

Fluxes of NO were measured using a closed box flux technique[15]. Prior to making flux measurements at each site, PVC cylindrical frames covering an area of 0.07 m$^2$ were driven 2 cm into the forest floor or soil. Flux measurements were made monthly in two of the three plots/N treatment/site (CP and CAO), with six flux boxes per plot. The procedure employs real time measurement of NO in the field using a Luminox nitrogen dioxide ($NO_2$) detector (Model LMA-3, Scintrex-Unisearch, Toronto, Canada) as described by Anderson and Poth[15].

## RESULTS AND DISCUSSION

### N Mineralization and Nitrification and N in Soil Solution

At CP total annual net nitrification (50 kg N ha$^{-1}$) in the soil plus litter was 4.5 times greater than at CAO (11 kg N ha$^{-1}$). At CAO, annual net nitrification in the forest floor and top 10 cm of soil (11 kg ha$^{-1}$) equaled 65% of the net N mineralization (17 kg ha$^{-1}$). At CP, annual net nitrification in the forest floor and top 10 cm of soil (50 kg ha$^{-1}$) equaled 76% of the net N mineralization (66 kg ha$^{-1}$). In soil, a higher percentage of the N mineralized was nitrified (67% at CAO and 91% at CP) than in the litter (50% at CAO and 43% at CP; Fig. 1). These results support the hypothesis that nitrification is a dominant process in the soils of these forests, even in the early stages of N saturation, and that rates are greater as N saturation progresses. This is particularly

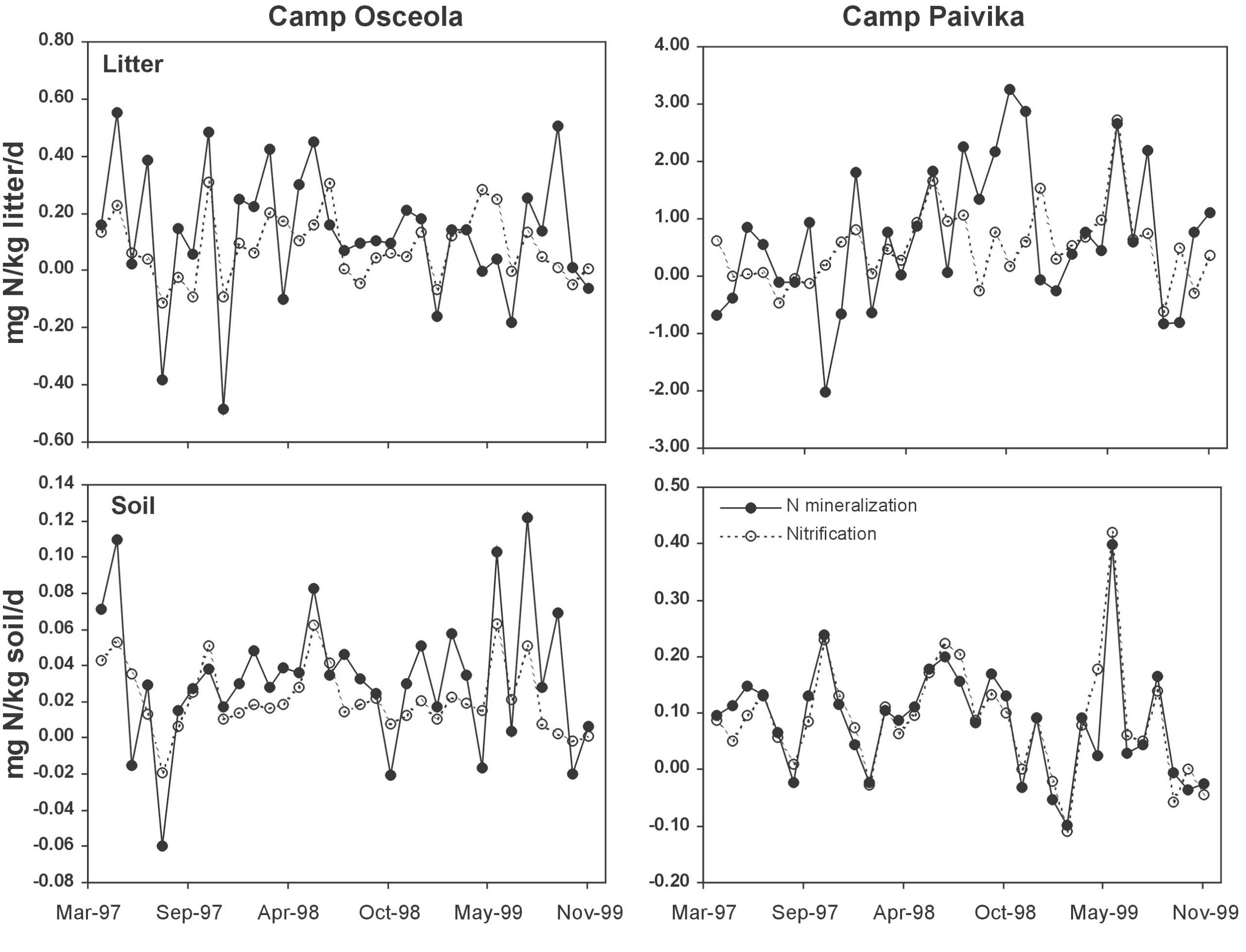

**FIGURE 1.** Net N mineralization and net nitrification rates in soil and litter in soil tubes incubated *in situ* at a N-saturated site (Camp Paivika) and an N-limited site (Camp Osceola) in the San Bernardino Mountains.

**TABLE 1**
**Nutrient Fluxes and Pools that are Affected by N Deposition in the San Bernardino Mountains**

| Process or Flux | Direction of Response to N Deposition | Comments |
|---|---|---|
| Net nitrification in mineral soil | ↑ | Virtually complete nitrification of mineralized N in high N deposition sites |
| Soil solution nitrate | ↑ | Levels commonly exceed 3000 μmol $l^{-1}$ |
| Nitrate leaching | ↑ | High nitrate levels in soil leachate and in streamwater |
| NO fluxes | ↑ | Rates are similar to fluxes from agricultural fields |
| C:N ratio of foliage, litter and soil | ↓ | |
| Litter production | ↑ | Due to increased N fertility and ozone-induced foliar abscission |
| Initial stage of litter decomposition | ↑ | |
| Long-term litter decomposition | ↓ | |
| Litter accumulation | ↑ | A thick forest floor mat occurs at sites in the western SBM |

*Note*: Nitrification is the central process controlling many of these responses and which leads to open or "leaky" N cycling.

true in the mineral soil where, on an areal basis (e.g., kg $ha^{-1}$), a larger fraction of net N mineralization and nitrification occur compared to the litter layer. Preliminary results of gross nitrification measurements from isotope pool dilution experiments also demonstrate that gross nitrification rates in soil at CP are dramatically greater than at CAO. As a result of nitrification, N is converted to the highly mobile nitrate form, contributing to open N cycling. High nitrification rates also result in a major increase in NO production in these forest soils, which is another avenue of N loss from these N saturated stands (Table 1). N cycling in undisturbed and low-pollution mixed conifer forests of California, as in most temperate forests, is normally highly conservative in retaining N, except for losses during periodic forest fires[16].

From this and a previous laboratory assay of net N mineralization and nitrification[9], it is clear that nitrification in litter lags behind N mineralization—by about 50% in the *in situ* incubations used in this study (Fig. 1). The factors which partially inhibit nitrification in litter are not known, but may be a result of the more acidic environment of the litter compared to the mineral soil, and/or because of inhibitory compounds in the litter[17,18].

The percentage of ammonium nitrified in soil was lower at CAO than at CP, but the percentage was still relatively high. This suggests that nitrification is an active process in California soils, even with relatively low levels of N deposition as evidenced by previous studies from various low pollution wildland ecosystems in California[19,20,21,22,23,24]. Although N deposition at CAO is approximately 5 to 7 times lower than at CP, levels are still above background. This raises the question as to whether the low but significant levels of N deposition at CAO might also stimulate nitrification rates. Studies are ongoing at mixed conifer sites along a north-south deposition gradient beginning in northern California where N deposition levels are near background levels, in order to test this possibility.

The Fernow Experimental Forest in West Virginia may be the most severely N-saturated site in the eastern U.S.[3,25], and the annual net nitrification rate (115 kg N $ha^{-1}$ $year^{-1}$)[26] is greater than at CP (41 kg N $ha^{-1}$ $year^{-1}$ in the mineral soil), but the percent N mineralized in soil which is nitrified is greater than 90% at both sites[25,26,27]. These findings suggest that the net nitrification:N-mineralization ratio may be effectively used, in combination with other data, as an index of site N status or severity of N saturation. Robertson[28] referred to this ratio as "relative nitrification," and suggested that this measure of nitrate production relative to the ammonium supplying capacity of the soil could allow for more meaningful cross-system and cross-site comparisons. The usefulness of this ratio as an N-saturation indicator needs to be verified at a greater number of sites under varying forest conditions and levels of N deposition in order to evaluate its general applicability. However, it appears likely that systems that have inherently high relative nitrification ratios will be prone to high losses of nitrate and gaseous N emissions under conditions of high N deposition. Ecosystems with a Mediterranean climate such as in California may be especially prone to nitrate leaching due to high runoff in the winter wet season when plant and microbial N demand is temperature restricted.

Ammonium concentrations in the soil solution were relatively low (peak concentration of 68 µEq $l^{-1}$) and were similar at CP and CAO. Nitrate concentrations ranged from 113 to 195 µEq $l^{-1}$ at CAO, but were 1679 to 4624 µEq $l^{-1}$ at CP (Fig. 2). These results corroborate the net nitrification results, demonstrating the dominance of nitrate in N cycling at CP.

### NO Emissions

In chaparral and mixed-conifer forests of southern California, NO flux rate from soil is an indicator of N saturation or site N status. Emissions from soil of $N_2O$ on the other hand are several-

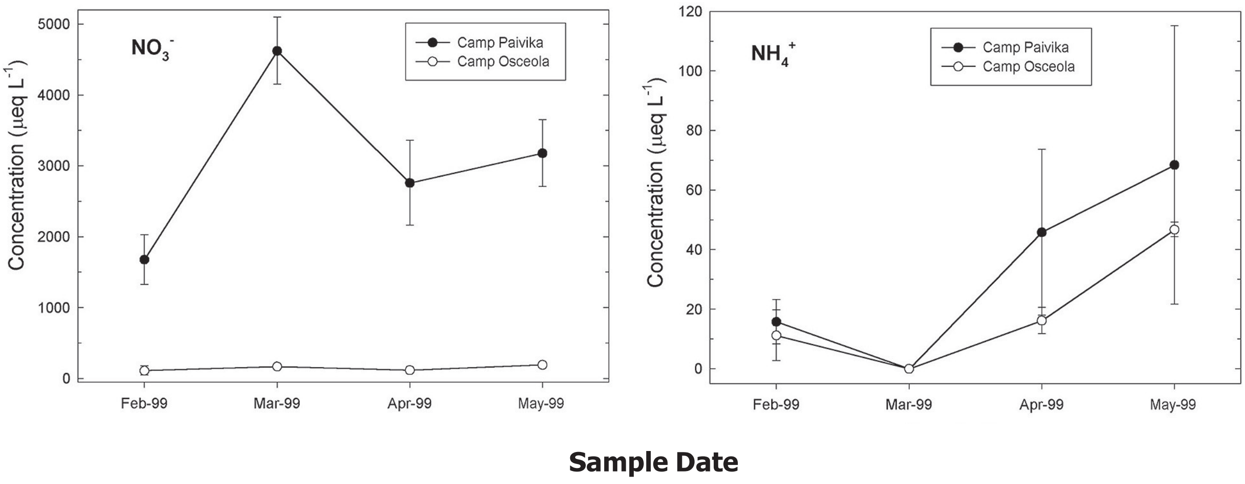

**FIGURE 2**. Concentrations of nitrate and ammonium in soil solution at Camp Paivika and Camp Osceola.

fold lower than NO fluxes in the upland areas of these systems[9,15]. Average annual fluxes of NO for the first 2 years of this study were 2.8 and 0.5 kg ha$^{-1}$ at CP and CAO, respectively. The fertilization treatments nearly always increased NO fluxes at CAO during periods of peak flux rates, while at CP (N-saturated site) flux rates were sometimes greater in the unfertilized plots (Fig. 3). These results support the hypothesis that N was often not limiting the production of NO at CP, but greater N availability in the N amended plots at CAO (N-limited site) led to greater NO emissions.

NO production in these coarse textured aerobic soils is putatively produced via nitrification. Fluxes of NO at CP are among the highest reported for temperate forests[29]. The much greater hydrologic export of nitrate[8] and gaseous losses of N from soil at CP, compared to the N-limited sites in the eastern SBM (e.g., CAO and BF), indicates the high degree of N excess or N saturation at CP. Trace gas emissions from soil of undisturbed upland forests with low N deposition inputs typically constitute a small and rather insignificant component of the annual N budget.

Trace gas emissions of excess N, at least in N saturated watersheds, are likely a much more significant N budget fraction in the riparian zone where moisture conditions are favorable for high nitrification and denitrification activity. This has not been studied in the SBM. However, in support of this hypothesis, we measured very high levels of dissolved $N_2O$ in water collected from a spring in Devil Canyon, the watershed that drains the CP region (M.A. Poth, and M.E. Fenn, unpublished data).

### Tree Growth Responses

After 4 years of N fertilization, bole-diameter growth of oak and pine did not respond to fertilization at CAO, while oak growth at CP was significantly greater in the 50 and 150 kg N ha$^{-1}$ treatments compared to the control. A parallel trend was observed in the pine trees at CP, but the increase was not statistically significant due to high variability in the response (Fig. 4). These findings were contrary to our expectations. It is not clear why tree growth did not increase at CAO and even decreased significantly in the oak trees fertilized with 150 kg N ha$^{-1}$ year$^{-1}$, but water limitation is one possibility. It will be important to follow the longer-term trends at both sites.

In previous short-term and high-dose fertilization experiments at CP (500 kg N ha$^{-1}$ as ammonium nitrate), foliar growth responses were negative or not significant[30]. In the current study, it was particularly surprising that trees responded to N fertilization at CP where nitrate concentrations in soil are extremely high during the growing season, the C:N ratio of soil is low, and foliar N levels and N:P ratios in foliage are significantly greater than at CAO. We can speculate that notwithstanding the high N fertility at CP, the N fertilization treatments may have provided plant available N during critical periods of plant N demand when available N may have been temporarily deficient. In recent studies at CP it was reported that carbon allocation belowground and fine root biomass in ponderosa pine were dramatically lower than at moderate and low-deposition sites[5,6]. These findings suggest that at CP, added N results in increased biomass accumulation in the bole as C transport belowground and assimilation by the roots is inhibited by ozone or N, or the combination of both pollutants.

### CONCLUSION

N cycling is highly open in nature (“leaky”) at CP in the western SBM. This is thought to be due to the dominance of nitrification in these forests as illustrated by the high ratio of nitrate produced relative to ammonium in net mineralization and net nitrification assays. The high nitrate:ammonium ratios in the soil solution also support these findings. Elevated nitrification rates result in high

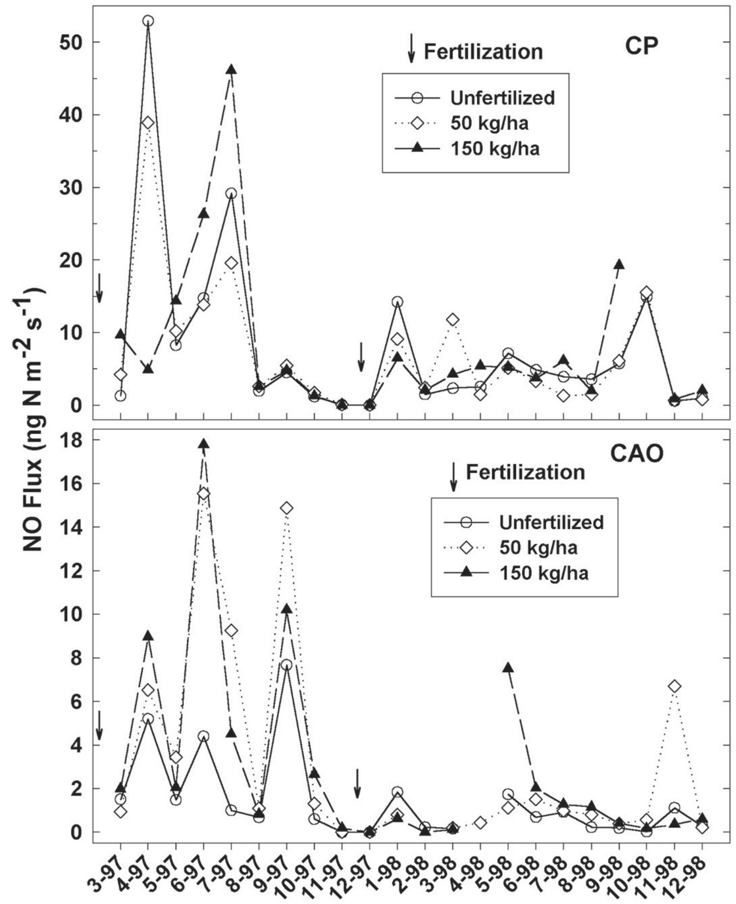

**FIGURE 3**. Nitric oxide (NO) fluxes from soil (*in situ*) at Camp Paivika (CP) and Camp Osceola (CAO) in plots receiving fertilizer treatments of 0, 50, or 150 kg N $ha^{-1}$ $year^{-1}$.

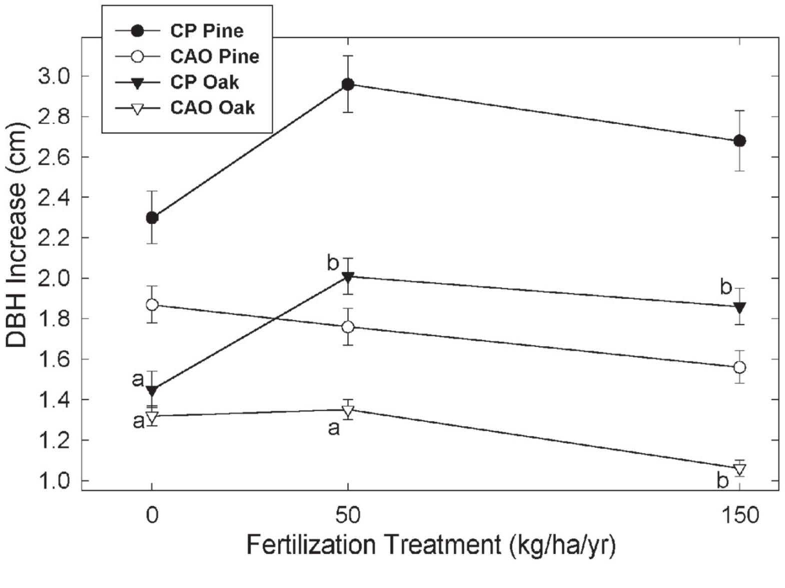

**FIGURE 4**. Bole growth increases in ponderosa or Jeffrey pine trees and California black oak trees after 4 years of N fertilization at Camp Paivika (CP) and Camp Osceola (CAO). Letters above the data points indicate significant differences between fertilizer treatment levels.

NO emissions from soil, further contributing to open N cycling. High hydrologic flows in the winter off-season when biological N demand is limited by low temperatures are another important characteristic leading to high nitrate losses from these systems. Further studies are needed to determine why tree diameter growth responded positively to added N at CP, when high N export rates demonstrate an excess of inorganic N.

| | | |
|---|---|---|
| Received: | July | 16, 2001 |
| Revised: | September | 23, 2001 |
| Accepted: | October | 5, 2001 |
| Published: | November | 8, 2001 |
